# Infection routes matter in population-specific responses of the red flour beetle to the entomopathogen *Bacillus thuringiensis*

**DOI:** 10.1186/1471-2164-15-445

**Published:** 2014-06-07

**Authors:** Sarah Behrens, Robert Peuß, Barbara Milutinović, Hendrik Eggert, Daniela Esser, Philip Rosenstiel, Hinrich Schulenburg, Erich Bornberg-Bauer, Joachim Kurtz

**Affiliations:** Institute for Evolution and Biodiversity, University of Münster, Hüfferstr. 1, 48149 Münster, Germany; Institute of Clinical Molecular Biology, Christian-Albrechts University Kiel, Schittenhelmstr. 12, 24105 Kiel, Germany; Zoological Institute, Christian-Albrechts University Kiel, Am Botanischen Garten 1-9, 24118 Kiel, Germany

**Keywords:** *Tribolium castaneum*, *Bacillus thuringiensis*, Host-parasite interactions, Oral infection, Pricking infection, RNA sequencing, Transcriptome

## Abstract

**Background:**

Pathogens can infect their hosts through different routes. For studying the consequences for host resistance, we here used the entomopathogen *Bacillus thuringiensis* and the red flour beetle *Tribolium castaneum* for oral and systemic (i. e. pricking the cuticle) experimental infection. In order to characterize the molecular mechanisms underpinning the two different infection routes, the transcriptomes of beetles of two different *T. castaneum* populations – one recently collected population (Cro1) and a commonly used laboratory strain (SB) – were analyzed using a next generation RNA sequencing approach.

**Results:**

The genetically more diverse population Cro1 showed a significantly larger number of differentially expressed genes. While both populations exhibited similar reactions to pricking, their expression patterns in response to oral infection differed remarkably. In particular, the Cro1 population showed a strong response of cuticular proteins and developmental genes, which might indicate an adaptive developmental flexibility that was lost in the SB population presumably as a result of inbreeding. The immune response of SB was primarily based on antimicrobial peptides, while Cro1 relied on responses mediated by phenoloxidase and reactive oxygen species, which may explain the higher resistance of this strain against oral infection.

**Conclusions:**

Our data demonstrate that immunological and physiological processes underpinning the two different routes of infection are clearly distinct, and that host populations particularly differ in responses to oral infection. Furthermore, gene expression upon pricking infection entailed a strong signal of wounding, highlighting the importance of pricking controls in future infection studies.

**Electronic supplementary material:**

The online version of this article (doi:10.1186/1471-2164-15-445) contains supplementary material, which is available to authorized users.

## Background

The route by which pathogens infect their hosts can have important consequences for host-pathogen interactions [1]. For example, a recent meta-analysis showed that virulence was higher in pathogens infecting wounded skin, compared with those ingested or inhaled [2]. Pathogens that can infect through alternative routes are particularly interesting systems with which to test the evolutionary and physiological consequences of infection through different routes. A recent study investigated the evolution of resistance of *Drosophila melanogaster* hosts against *Pseudomonas entomophila* bacteria upon oral as compared to systemic (i. e. pricking the cuticle) infection [3]. Interestingly, hosts evolved resistance towards the bacteria for both routes of infection. However, there was no cross-resistance, i. e. *D. melanogaster* selection lines that had evolved resistance against *P. entomophila* upon oral infection were not more resistant against *P. entomophila* upon pricking and vice versa. This route-specificity indicates that the physiological underpinnings of resistance and therefore the evolutionary trajectories of adaptation differ for the routes of infection. However, it is currently unclear whether such route-specificity is a general phenomenon or restricted to this particular host-pathogen system.

In the present study, we studied infection route-specificity in the red flour beetle *Tribolium castaneum* upon oral and pricking infection with the entomopathogen *Bacillus thuringiensis* [4, 5]. We compared gene expression between pricking and oral infection, using an Illumina next generation sequencing approach (RNA-seq). RNA-seq is a powerful tool that enables a very precise quantification of transcript levels on a genome-wide scale [6]. For our comparative RNA-seq based study, a sterile wounding treatment was included to distinguish between effects of wounding alone and bacterial infection.

*T. castaneum* is a relevant global stored product pest that has developed into a fully-fledged insect model organism [7]. The genome of the *T. castaneum* strain Georgia 2 (GA-2) has been fully sequenced in 2008 [8]. *B. thuringiensis* is a Gram-positive bacterium that forms highly resistant endospores and plasmid-encoded crystalline inclusions (Cry proteins), which are toxic upon oral ingestion [9]. Cry toxins provide specificity for insect orders upon oral infection [10]. We recently showed that spore-crystal mixtures of the *B. thuringiensis bv.tenebrionis* strain are infectious to *T. castaneum* upon oral exposure [4]. However, *B. thuringiensis* is also able to efficiently infect and kill *T. castaneum* upon pricking infection [5]. Insects may regularly suffer from wounding in their natural environments, such that infection through the wounded cuticle is likely to occur in nature. Moreover, septic pricking in the laboratory can lead to spore production in cadavers (unpublished data). Defense of *T. castaneum* against septic infection with *B. thuringiensis* has been studied as a model for eco-immunology and the host has been shown to be capable of specific immune priming within and across generations [5, 11]. Recently, it was also shown that the oral exposure to *B. thuringiensis* spore supernatants leads to priming of *T. castaneum* larvae. The analysis of the host responses to oral and pricking infection may contribute to an enhanced understanding of the mechanistic underpinnings of this astonishing degree of immunological adaptation.

Natural variation in resistance may provide a basis for studying evolved differences among host populations, and thereby provides a potentially important source of information for the identification of the genetic causes of resistance. We have previously shown that a recently collected population of *T. castaneum* (Cro1) showed enhanced resistance to oral infection with *B. thuringiensis*, as compared to commonly used laboratory populations (SB, GA-2) [4]. In the present study, we therefore compared gene expression between the populations Cro1 and SB upon oral and pricking infection with *B. thuringiensis*. We found a higher number of differentially expressed genes in the Cro1 population for both routes of infection. Intriguingly, gene expression profiles differed strongly between the oral and pricking routes of infection, indicating that immunological and physiological processes underpinning the two routes of infection are clearly distinct. Furthermore, we demonstrate that pricking without bacteria, i. e. a sterile aseptic wounding, leads to strong immune activation and therefore represents a necessary control for pricking infections.

## Results

### Single-nucleotide resolution transcriptome of *T. castaneum*by RNA-seq

**Figure 1:**
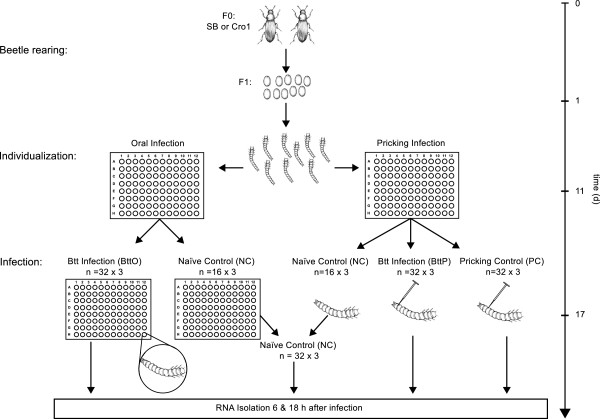
**Schematic illustration of the experimental design.** Approximately 500 two week old adult *T. castaneum* beetles (population SB or Cro1) were reared under standardized conditions (30°C, 60% relative humidity, 12/12 h light/dark cycle). After 24 h of egg-laying, eggs were separated from beetles and further incubated. Hatched larvae were then individualized into 96’-well plates and assigned to either oral or pricking challenge with *B. thuringiensis*. Naïve controls from oral and pricking infection were pooled upon RNA isolation. For RNA isolation, 32 larvae were pooled for one replicate per treatment. In total, three replicates for each treatment were used which results in a total number of 1,536 animals that were used for this study (32 larvae × 4 treatments × 2 populations × 2 time points × 3 replicates).

RNA-seq experiments were performed for two different *T. castaneum* populations, SB and Cro1, both at 6 h and 18 h after infection with *B. thuringiensis*. Two different infection routes were applied: pricking infection (BttP) and oral infection (BttO). Furthermore, a pricking control (PC) as well as a naïve control (NC) were included; for an overview of the experimental design see Figure 1. Each treatment was replicated three times resulting in a number of 48 samples. In total, 2.9 billion paired-end reads were obtained and after filtering 2.4 billion reads were mapped against the *T. castaneum* reference genome. Of these, 1.7 billion reads (around 70%; see Additional file 1) could be aligned to the *T. castaneum* genome yielding 170 billion bp sequence information and >4000× coverage of the *T. castaneum* transcriptome. Afterwards every single treatment (BttO, BttP and PC) was tested for differential expression against the corresponding NC for a fixed population (SB and Cro1) and a fixed time point after exposure (6 h and 18 h) resulting in twelve pairwise comparisons. The numbers of differentially up- and downregulated genes for every pairwise comparison are summarized in Additional file 2. In order to examine the degree of variation in the replicate expression profiles, principal component analyses were performed for the two time points separately. In the PCA for the 6 h time point, the first component explains 43% of the variability and the second 36%, for the 18 h time point 59% and 21% respectively. As can be observed in Additional files 3 and 4, the replicates cluster together indicating a high reproducibility of the experiment.

### Numbers of differentially expressed genes differ between populations and infection routes

**Figure 2:**
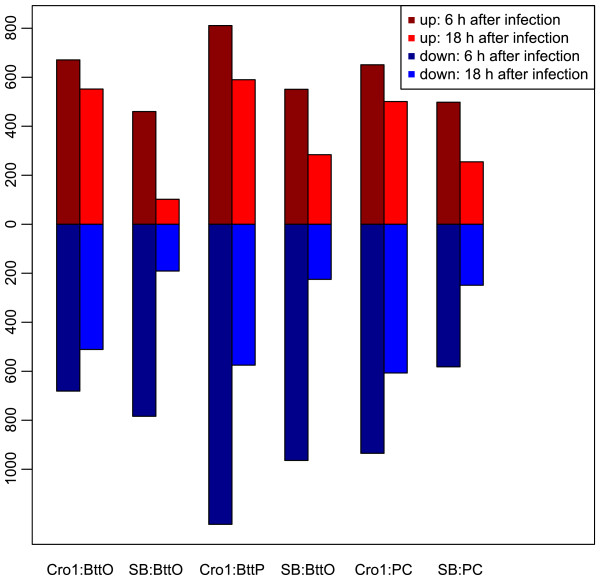
**Numbers of differentially expressed genes 6 and 18 h after infection.** The upper bars show the number of significantly upregulated genes, the lower bars the number of significantly downregulated genes for all combinations of the two populations Cro1 and SB and the three different treatments BttO, BttP and PC against their respective naïve controls. Significant up- and downregulation is based on Cufflinks analyses with the default q-value cutoff of 0.05 [60]; see Additional file 2.

**Figure 3:**
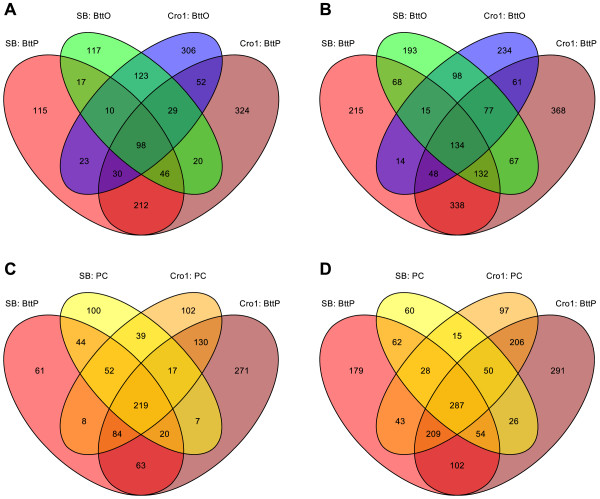
**Venn diagrams of differentially expressed genes 6 h after infection.** The sets of differentially expressed genes result from differential expression analyses for every treatment against its naïve control using Cufflinks with the default q-value cutoff of 0.05 [60]. Venn diagram of significantly **A)** upregulated genes in all combinations of the populations Cro1 and SB and the treatments BttO and BttP, **B)** downregulated genes in all combinations of the populations Cro1 and SB and the treatments BttO and BttP, **C)** upregulated genes in all combinations of the populations Cro1 and SB and the treatments BttP and PC, **D)** downregulated genes in all combinations of the populations Cro1 and SB and the treatments BttP and PC.

When comparing the numbers of differentially expressed genes between the two different time points 6 h and 18 h after exposure (see Additional file 2), applying Wilcoxon rank-sum test reveals that significantly more genes are differentially expressed 6 h after infection compared to 18 h after infection (*p*=0.001). This behavior can also be observed in Figure 2 showing that this trend holds both true for numbers of up- and downregulated genes. Furthermore, for every treatment compared against NC, Cro1 shows a higher number of differentially expressed genes than SB (for BttO: *p*=0.013; for BttP and for PC: *p*<2.2·10^−16^; Fisher’s Exact Test). Figure 3 reveals that of the genes that are significantly upregulated in either BttO or BttP 6 h after infection, only 16.36% are specific to SB while 44.81% are Cro1-specific and 38.83% are population-unspecific. Following a less extreme but yet similar trend, of the genes that are significantly downregulated in either BttO or BttP 6 h after infection, only 23.08% are SB-specific, 32.15% are Cro1-specific and 44.76% are population-unspecific. The reaction to BttO compared to BttP 6 h after infection is more population-specific (for upregulation: *p*=7.7·10^−6^; for downregulation: *p*=4.2·10^−14^; Fisher’s Exact Test) implying that the population difference between Cro1 and SB is higher with respect to oral infection. Moreover, as can be observed in Figure 3, the overlap between BttP and PC compared to BttP and BttO 6 h after exposure is larger for both significantly up- and downregulated genes (for both up- and downregulation: *p*<2.2·10^−16^; Fisher’s Exact Test). The same trends apply for the 18 h after infection treatments; see Additional file 5.

### Cuticle genes are highly enriched among differentially expressed genes

**Figure 4:**
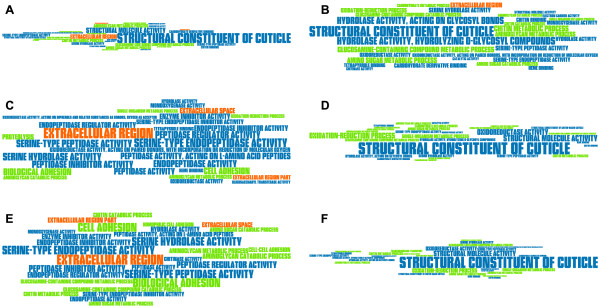
**TermLogos of overrepresented GO terms in sets of differentially expressed genes.** GO terms are based on Blast2GO and InterProScan, the scaling factor for the font size is given by *S*=| log10(p-value)| where the p-value corresponds to the p-value of the overrepresentation analysis (Fisher’s Exact Test) that was corrected for multiple testing (Benjamini-Hochberg; p-value cutoff 0.05). TermLogos visualize overrepresented GO terms such that the font size represents the significance of every GO term: the larger the font, the smaller the corresponding p-value [12]. Only the top 30 GO terms are displayed. The panels show overrepresented GO terms in significantly **A)** upregulated genes in BttO, **B)** downregulated genes in BttO, **C)** upregulated genes in BttP, **D)** downregulated genes in BttP, **E)** upregulated genes in PC and **F)** downregulated genes in PC; color code: orange - GO domain “cellular component”, blue – GO domain “biological process”, green – GO domain “molecular function”; for a list of the TOP 30 overrepresented GO terms and their corresponding p-value see also Additional file 6.

In order to detect Gene Ontology (GO) terms that are overrepresented in a gene subsample of interest against all *T. castaneum* genes as background, we applied Fisher’s Exact Test with a multiple testing corrected p-value cutoff of 0.05 and generated a TermLogo similar to [12]. Most prominently, as can be observed in Figure 4, the GO term “structural constituent of cuticle” shows up in 5 of the 6 TermLogos. Strinkingly, this GO term is significantly overrepresented in the significantly upregulated genes of the BttO samples (*p*=7.8·10^−41^) while it seems to be of minor importance among the overrepresented GO terms for the significantly upregulated genes in the BttP and PC samples (for BttP *p*=0.028, for PC *p*>0.05). By contrast, this GO term appears to be highly enriched among the significantly downregulated genes of the BttP and PC samples (for BttP *p*=5.6·10^−55^, for PC *p*=3.5·10^−55^) while for BttO, the enrichment is not as high for significantly down- as compared to upregulated genes, but still highly significant (*p*=2.5·10^−22^). Furthermore, in accordance with our previous findings, Figure 4 reveals more similarities between the two pricking treatments BttP and PC and shows a clear distinct GO term enrichment profile for BttO. Especially we see an enrichment of GO terms related to serine-type peptidases, endopeptidases and endopeptidase inhibitors among significantly upregulated genes of the two pricking treatments. The latter finding is consistent with a recent RNA-seq study that also revealed these GO terms as overrepresented in samples of *Tenebrio molitor* beetles that were injected with heat-killed *Staphylococcus aureus* bacteria [13]; for a list of the TOP 30 overrepresented GO terms per treatment and their corresponding p-value see Additional file 6.

### Populations show specific patterns of gene regulation

**Figure 5:**
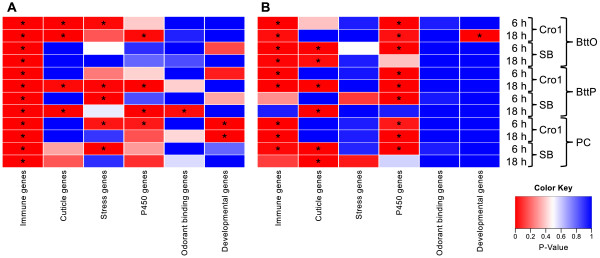
**P-value heatmaps for immune, cuticle, stress, P450, odorant binding and developmental genes.** P-values are based on the overrepresentation analysis of defined gene sets (immune gene set [14], developmental gene set [8] and other gene sets based on annotation downloaded from the EnsemblMetazoa database, release 17 [65]) in the lists of significantly **A)** upregulated and **B)** downregulated genes for every of the twelve pairwise differential expression analyses; an asterisk indicates *p*<0.05 (Fisher’s Exact Test; p-value corrected for multiple testing according to the Benjamini Hochberg procedure). Significant up- and downregulation is based on Cufflinks analyses with the default q-value cutoff of 0.05 [60].

To investigate whether the up- or downregulation of immunity-related genes plays a fundamental role in response to BttO, BttP or PC, we tested whether immune genes (a set of genes based on [14]; see Additional file 7) are significantly overrepresented in the lists of significantly up- or downregulated genes for every pairwise differential expression result using Fisher’s Exact Test. The resulting p-values which were corrected for multiple testing according to the Benjamini Hochberg procedure are depicted in Figure 5. This heatmap clearly reveals that immune genes are significantly overrepresented both in the lists of significantly upregulated genes (12/12 lists) as well as in the lists of significantly downregulated genes (9/12 lists) genes.

In order to further investigate the enrichment of the GO term “structural constituent of cuticle” in sets of differentially expressed genes described in the preceding paragraph, we also applied the overrepresentation analysis to a list of cuticle genes, i. e. to a list of all genes with the GO term “structural constituent of cuticle”; see Additional file 8. For BttP, Figure 5 shows a similar pattern in the two different populations: cuticle genes are highly enriched 18 h after infection in Cro1 and SB in both significantly up- and downregulated genes. For PC, we observe an enrichment of cuticle genes only in the lists of significantly downregulated genes of the SB treatments. Noticeably, Figure 5 demonstrates that cuticle genes are highly enriched in the significantly upregulated genes 6 and 18 h after oral infection in Cro1 (and not in the downregulated genes) while for SB, they are significantly overrepresented in the significantly downregulated genes 6 and 18 h after oral infection (and not in the upregulated genes). This clearly reveals another population-specific reaction upon oral infection: for Cro1, relatively more cuticle genes are up- than downregulated and for SB, relatively more cuticle genes are down- than upregulated.

A recent transcriptome study of *T. castaneum* hypothesized a crosstalk between stress-related genes, cytochrome P450s and between immune genes [15]. This is why we defined a set of “stress genes” (see Additional file 9) and another set of “P450 genes” (see Additional file 10) and tested for enrichment of these gene sets in our differential expression results. Figure 5 shows that stress genes are significantly overrepresented in the lists of significantly upregulated genes (5/12 lists) with a slightly stronger enrichment in Cro1 compared to SB. Thus, this supports the hypothesis of an interdependency between stress and immunity related genes. Remarkably, we observe a very strong overrepresentation of cytochrome P450s in the lists of significantly downregulated genes (9/12 lists). Hence, we suggest that downregulation of cytochrome P450 genes plays an important role in response to all three treatments BttO, BttP and PC. Interestingly, for Cro1, P450 genes are enriched in the 6 h as well as 18 h after exposure treatments, while for SB, P450 genes are only enriched in the 6 h after exposure treatments. Thus, we hypothesize a prolonged downregulation of cytochrome P450s in Cro1 compared to SB.

Upon oral infection, many odorant binding proteins are found differentially regulated (see Additional file 11). Odorant binding proteins have been associated to host defense and there is evidence that they play a role in resistance to *B. thuringiensis* intoxication in *T. castaneum* [16]. For this reason a further set of “odorant binding genes” was defined; see Additional file 12. As can be observed in Figure 5, odorant binding genes are only enriched among significantly upregulated genes in SB, 18 h after pricking infection. Nevertheless, we found several odorant binding genes differentially expressed in BttO, BttP and PC. Moreover, each population expresses a different set of odorant binding genes. For example, 6 h after oral infection, *OBP-C04*, *OBP-10* and *OBP-C12* are significantly upregulated in Cro1 while *OBP-8*, *OBP-12* and *OBP-19* are significantly upregulated in SB. For *OBP-C12*, a role in the defense of *T. castaneum* has been already described in [16].

A study in *T. castaneum* conjectured an interaction between immune and developmental genes [17]. Therefore, the overrepresentation analysis was also applied to a list of developmental genes taken from [8]; see Additional file 13. As can be observed in Figure 5, the category “developmental genes” is significantly overrepresented in the significantly upregulated genes of the Cro1 samples BttP, 6 h after infection, PC, 6 h and 18 h after exposure. Furthermore, these developmental genes are enriched in the significantly downregulated genes of the Cro1 sample BttO, 18 h after infection. Remarkably, the developmental genes are not enriched in any of the SB samples, neither for up- nor for downregulated genes. Thus, up- and downregulation of developmental genes upon oral infection, pricking infection and pricking control seem to constitute an important response mechanism of the beetle population Cro1 while for SB, the involvement of developmental genes in response to these types of exposures appears to be negligible.

### Immune gene categories show population-specific differential regulation

**Figure 6:**
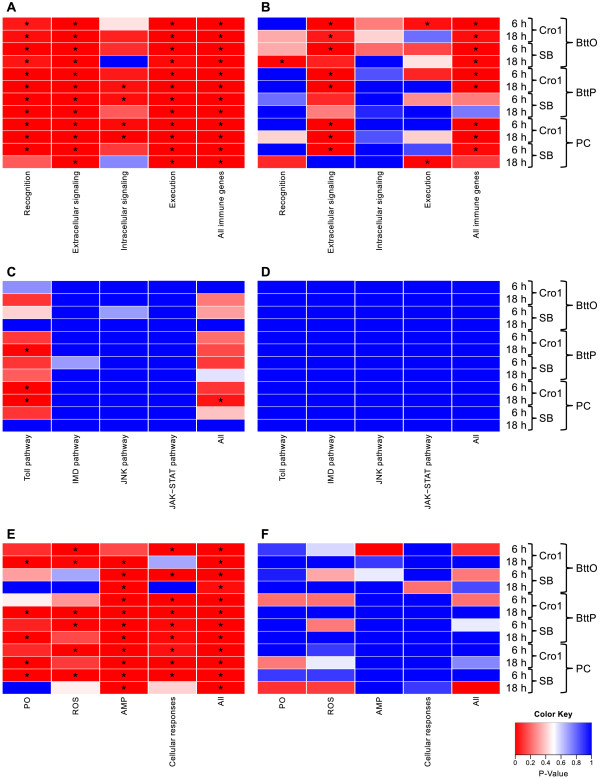
**P-value heatmaps for immunity-related categories.** Immune genes were separated into the categories “recognition”, “extracellular signaling”, “intracellular signaling” and “execution” according to [14]. P-values are based on the overrepresentation analysis of **A)** immune gene sets in significantly upregulated genes, **B)** immune gene sets in significantly downregulated genes, **C)** “intracellular signaling” gene sets in significantly upregulated genes, **D)** “intracellular signaling” gene sets in significantly downregulated genes, **E)** “execution” gene sets in significantly upregulated genes and **F)** “execution” gene sets in significantly downregulated genes for every of the twelve pairwise differential expression analyses; an asterisk indicates *p*<0.05 (Fisher’s Exact Test; p-value corrected for multiple testing according to the Benjamini Hochberg procedure). Significant up- and downregulation is based on Cufflinks analyses with the default q-value cutoff of 0.05 [60].

To further analyze immunity-related genes, they were separated into the categories “recognition”, “extracellular signaling”, “intracellular signaling” and “execution” according to [14] (see Additional file 7). As can be observed in Figure 6, the categories “recognition”, “extracellular signaling” and “execution” play an important role in the lists of significantly upregulated genes. For downregulation, “extracellular signaling” seems to be the key player among the immune gene categories (8/12 lists). In order to investigate which immune pathways are involved in the response to oral infection, pricking infection and pricking control, we repeated the overrepresentation analysis for subcategories of “intracellular signaling” (“Toll Pathway”, “IMD pathway”, “JNK pathway” and “JAK-STAT pathway”, see Figure 6, III and IV) and subcategories of “execution” (with subcategories “Phenol Oxidases (PO)”, “Reactive Oxygen Species (ROS)”, “Antimicrobial Peptides (AMP)”, “cellular responses”, see Figure 6, V and VI). For downregulated genes, neither the “intracellular signaling” nor the “execution” subcategories are overrepresented in any of the treatments. However, for upregulated genes the enrichment analysis reveals the following. The only overrepresented “intracellular signaling” pathway is the “Toll pathway” which remarkably solely shows a significant enrichment for some particular Cro1 samples and for none of the SB samples (see Figure 6, III). All “execution” subcategories are significantly overrepresented in the significantly upregulated genes of at least 5 of 12 treatments (see Figure 6, V). Noticeably, for 9 of the 11 treatments, significantly many of the upregulated genes fall into the subcategory “cellular responses”. Most strikingly, for 11 of 12 treatments we observe an enrichment of AMPs in the upregulated genes. Thus, for both beetle populations, oral infection, pricking infection as well as pricking control result in an upregulation of AMPs both 6 h and 18 h after exposure. The overrepresentation analysis of “execution” subcategories for upregulated genes further reveals the following population difference: for Cro1, PO and ROS show a significant overrepresentation 6 h after oral infection and AMP does not, while for SB, AMPs are enriched in the upregulated genes 6 h after oral infection and PO and ROS are not. Hence, the predominant execution mechanisms induced by oral infection seem to differ in the two populations 6 h after infection: PO and ROS for Cro1, AMPs for SB. By contrast, the overrepresentation analysis of execution mechanisms in upregulated genes does not unveil any noticeable population difference for the pricking infection and pricking control samples. This is in line with our previous finding based on numbers of differentially expressed genes: the reaction to oral infection compared to pricking infection is more population-specific.

**Figure 7:**
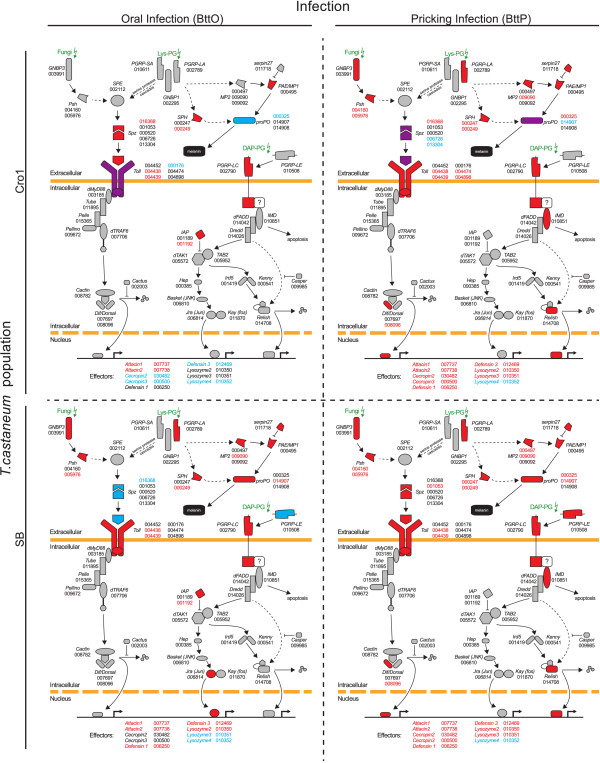
**Regulation of the Toll and IMD pathway for BttO and BttP 6 h after infection.** Illustrated are the Toll and IMD pathways after [14] for the two populations of *T. castaneum* Cro1 and SB and for the two infection methods BttO and BttP. Red indicates significant upregulation and blue significant downregulation of respective genes. The corresponding official gene IDs (’TC######’) are specified next to the genes. When different gene family members are both significantly up- and downregulated, genes are presented in a purple manner and the colors of the corresponding official gene ID indicate in more detail which isoforms are up- and which ones are downregulated. Only effectors that are differentially expressed 6 h after infection in at least one treatment are indicated with their respective official gene IDs; see also Additional file 7.

A deeper investigation of gene regulation in the Toll and IMD signaling cascades reveals further differences between infection routes and beetle populations; see Figure 7. While pricking infections lead to similar expression patterns in the IMD and Toll pathways, the oral infection route shows a more distinct pattern for each beetle strain, especially in the Toll regulated PO cascades and in the expression of effector molecules (AMPs). While AMP regulation upon pricking infection shows the same trends for Cro1 and SB, the expression of AMPs upon oral infection is highly divergent between the two populations. When comparing infection routes 6 h after exposure, BttP follows a more “classical” mode of action than BttO: transcription factors (*Dif2*, *REL2* (Relish)) for both pathways are significantly upregulated upon pricking infection while orally infected beetles show no differential expression of these transcription factors; see Figure 7. Additionally, the expression of JAK-STAT regulated effectors is very distinct for each infection route. For example, *TEP-A* and *TEP-C* are both significantly upregulated upon pricking infection, while only *TEP-C* is significantly upregulated upon oral infection (see Additional file 7). In sum, we observe striking differences in regulation of Toll and IMD pathways and of JAK-STAT regulated effectors between the two different routes of infection.

## Discussion

In this study, we compared for the first time host gene expression profiles for differing routes of infection with the same bacterium, *B. thuringiensis*. We found that the routes of infection, oral and systemic (by pricking the cuticle) induce strikingly different transcriptional profiles in the host. Secondly, by using two host populations, we could show that the host populations show specific differences in their transcriptional responses. Intriguingly, the factors infection-route and host population interact, such that population differences depend on the infection routes and were generally more pronounced for oral infection.

### Genetic underpinnings of infection route-specificity

A number of pathogens are able to infect by different routes, but in-depth analyses of global host response profiles when pathogens enter the body in different ways are as of yet lacking. Insects are important model organisms for studying responses to pathogen entry. Making primarily use of the fruit fly *Drosophila melanogaster*, injection or pricking of the cuticle for application of bacteria has been the method of choice that has enabled the identification of the main regulatory pathways of innate immunity [18–22]. More recently, protocols for oral infection with natural bacterial pathogens are increasingly being employed for studying gut immunity [23–26]. However, prior studies have focused on either way of infection, and as far as we are aware, no study has as of yet compared in detail the host responses to both ways of infection for the same pathogen. Transcriptome analysis (RNA-seq) was here used as a powerful tool to compare responses of the red flour beetle *T. castaneum* to oral and pricking (i. e. systemic) infection with *B. thuringiensis*. Strong differences in gene expression profiles were observed for both infection routes, at both an early (6 h) and a later (18 h) time point after infection. Essentially, the overlap of genes between oral and pricking infection was strikingly low, which clearly supports the recent findings that evolutionary adaptation of *D. melanogaster* to a bacterial pathogen is contingent upon the infection route [3]. It is particularly noteworthy that different host-pathogen systems were used in [3] and the present study, which points to the generality of the phenomenon of infection route-specificity. Moreover, it demonstrates that physiological responses (this study) and evolutionary trajectories [3] are connected, i. e. both types of studies are complementary.

In our study, vegetative cells were used for pricking, while spore toxin mixtures were used for oral infection. Although this might account for some differences in host gene expression, the specific processes following the two routes of infection are rather likely responsible for the strong differences. Opposite to systemic infection, *B. thuringiensis* infecting via the oral route has to use different strategies to enter the host. In contrast to the situation in the hemolymph, in the gut, *B. thuringiensis* has to prevent ejection by stopping or reducing peristalsis, and it has to breach the peritrophic membrane and gut epithelium. The different physiological consequences of oral and pricking infection are clearly represented in the strongly diverging GO term (Figure 4) and enrichment analyses (Figures 5 and 6). Pricking infection entails a strong signal for stress and cytochrome P450 genes, supporting a previous study [15]. Differences in infection routes are particularly obvious for immune genes. Pricking infection provides the expected immune gene activation, resulting in induction of Toll and IMD pathways (Figures 6 and 7) that lead to strong AMP expression. Moreover, further execution mechanisms such as ROS and cellular responses are activated. Similar trends were also observed in a recent RNA-seq study in which *Tenebrio molitor* beetles were injected with heat-killed *Staphylococcus aureus* bacteria [13]. By contrast, oral infection provides a more complex picture, where the two beetle populations showed surprisingly contrasting gene activation patterns, as will be outlined in more detail below.

### Host populations differ in expression profiles upon oral infection

We used two host populations for our comparison of infection-route specific gene expression profiles: a widely used laboratory strain (SB) and a newly collected strain (Cro1). The latter strain was previously shown to be more resistant to oral infection [4]. A comparison of gene expression upon oral infection may thus help to identify genetic underpinnings of the difference in resistance. Accordingly, we found much stronger transcriptional responses to infection in the Cro1 strain. Most interestingly, while the populations reacted rather uniformly to pricking infection, expression patterns of certain groups of genes showed markedly differing responses to oral infection. Enrichment of cuticular protein genes showed such population-specific patterns for oral infection (up-regulated in Cro1, while down-regulated in SB; Figure 5). By contrast, pricking did not lead to population-specific patterns for this class of genes. The strong pattern for cuticular protein genes could be a consequence of infection-induced developmental asynchrony compared to naive beetle larvae, since molting entails cuticular turnover, as suggested by [27], for honeybees 36 h after oral infection with *Paenibacillus larvae*, the cause of American Foulbrood Disease. However, this might be somewhat less likely in our study, since differences were observed already at an early time-point as short as 6 h post infection (p. i.). Accordingly, we found enrichment for down-regulated developmental genes in Cro1, but not in SB, only at the later time point of 18 h post oral infection (Figure 5). It is possible that Cro1 is more resistant because it shows developmental adjustment when exposed to *B. thuringiensis*. This hypothesis is supported by our recent demonstration of oral priming for resistance in *T. castaneum*, which also entailed developmental retardation [28]. By contrast, *T. castaneum* that were prick-primed with heat-killed bacteria were previously shown to speed up development and thereby potentially escape a perceived risk of infection [17]. This effect differed among beetle lines, which is supported by the observation in the present study that developmental genes were enriched among up-regulated genes in the pricking control in the Cro1, but not the SB population. It could be that the Cro1 population has retained an adaptive developmental flexibility that has been lost in the SB strain that was maintained in the laboratory for many generations, often subjected to a rigid developmental timing and in absence of relevant pathogen pressure. These differences among beetle lines are indicative of genetic variance in the interaction between immunity and development and are clearly a fascinating field of further investigation. Epigenetic regulation might connect immunity and development, as recently demonstrated for a lepidopteran host, the greater wax moth *Galleria mellonella* [29]. An alternative explanation for the relevance of genes with cuticular functions is the relevance of the peritrophic membrane for oral infection. This inner lining of the midgut contains chitin, and degradation of the peritrophic membrane is an important step during gut infection, since it enables bacteria to gain access to epithelial cells [30]. Peritrophic membrane repair and induction of chitin-specific enzymes can be expected during gut infection, and accordingly, chitin deacetylase was found up-regulated upon oral infection. Chitin deacetylases are important for chitin turnover in *T. castaneum* [31]. Moreover, in the context of infection, it might be potentially interesting that chitin deacetylation results in the formation of chitosan, a powerful antimicrobial agent [32, 33].

Based on oral infection of *D. melanogaster* with *Erwinia carotovora*, a study from 2009 suggests that gut homeostasis is maintained through a balance between cell damage due to the collateral effects of bacteria killing and the repair of the gut barrier, i. e. peritrophic membrane and epithelia [25]. Moreover, this analysis suggests that the IMD pathway directly participates in the remodeling of this barrier, while no evidence for the involvement of the Toll pathway in immune response against this oral infection was found [25]. Our findings support this view, however, the situation seems even more complex, because the two host populations showed different responses (Figure 7). SB shows a pattern that is mostly consistent with *D. melanogaster* infected with *E. carotovora*, i. e. an up-regulation of the IMD pathway and therefore several AMPs. However, SB beetles also showed up-regulation of some (but not all) components of the Toll pathway. By contrast, Cro1 does not show a clear up-regulation of the IMD pathway and even a down-regulation of some of the Toll receptors. This relates to a clearly distinct pattern of the two populations regarding effector functions involved in oral infection: SB basically shows an AMP-based response to oral infection, while Cro1 seems to rely on immunity provided by PO and ROS (Figure 6). Arguably, the latter type of response might be more efficient to clear an oral infection with *B. thuringiensis*, leading to increased resistance of the Cro1 population. ROS provide a very fast response to infection [34]. As the infection outcome of *B. thuringiensis* in *T. castaneum* is dose dependent [4], fast reduction of parasite load at the beginning of infection may indeed improve host survival. However, ROS may also lead to oxidative damage of self tissue and its need for renewal [25], i. e. there might be a cost the host has to pay for protection against infection. Accordingly, an enrichment among up-regulated genes was observed for stress genes 6 h p. i., followed by Cytochrome P450 (CYP) genes 18 h p. i., only in the Cro1, but not the SB population (Figure 5). CYP are a diverse class of enzymes with versatile functions including metabolic detoxification and resistance [35, 36]. The observed up-regulation of stress and CYP genes may thus function to alleviate immunity-related self-damage. Taken together, these data show that host populations may markedly differ in their way they deal with bacterial oral infections and its collateral damage. The SB population has been in the laboratory since many generations, likely with reduced exposure to pathogens, which might have led to the evolutionary loss of efficient, but costly immune strategies. Such rapid evolution is possible, as also demonstrated by [3]. The observed population differences in immunity to oral infection might also result from differences in the gut microbiota of the host populations. Gut microbiota have for example been shown to be involved in immune priming for resistance against *Plasmodium* in mosquitoes [37]. In this study, immune priming was related to responses of hemocytes to the natural gut microbiota that enters the hemocoel upon penetration of the gut epithelium by *Plasmodium* ookinetes. Since *B. thuringiensis* oral infection also damages the gut epithelium, it is not unlikely that part of the response to oral infection could be a response to gut microbiota. Potential differences in gut microbiota of the two study populations might even explain the observed differences in host responses to infection. Interestingly, gut microbiota have been suggested to be relevant in *B. thuringiensis* infections in lepidopteran hosts [38–40]. These alternative or complementary explanations will be a fascinating field for further research.

### Genes involved in oral infection with *B. thuringiensis*

A number of genes have previously been reported to be involved in the oral infection process of *B. thuringiensis* and are thus candidate resistance mechanisms. The Cry toxin of *B. thuringiensis* is the most intensively studied virulence factor. Of the reported Cry receptors [41, 42], several were found down-regulated upon oral exposure. Aminopeptidase N-like genes, E-cadherin (*TcCad1*) and sodium solute symporter protein (*TcSSS*) were down-regulated in both host populations at 6 h p. i. Although *TcCad1* and *TcSSS* were found to bind the *Cry3Ba* more strongly than the *Cry3Aa* toxin of the beetle-infective *B. thuringiensis* strain *tenebrionis* that was used here [42], its down-regulation in our experiment may nevertheless suggest a role in infection with *B. thuringiensis tenebrionis*. Apolipophorin III (*ApoLp-III*) gene transcripts were elevated upon oral infection. This gene has been involved in host defense in several insect species [43, 44] and was recently shown to participate in the regulation of hemolymph PO activity in larvae infected with *Cry3Ba* producing strain [42]. Further genes with a hypothesized role in defense against *B. thuringiensis* in *T. castaneum* were found up-regulated in our study [16, 42]. A variety of odorant binding proteins (OBPs) and chemosensory proteins (CSPs) were significantly up-regulated 6 h p. i. in both beetle populations. Generally, CSPs and OBPs are involved in olfaction and chemical communication in insects, but the functions of members of these large families of genes might be diverse [8, 45]. Elevated levels of CSP gene transcripts were previously reported after oral exposure to *B. thuringiensis* or its toxins in *Tenebrio molitor* [46] and *T. castaneum* [16], and a role of *OBP-C12* in *T. castaneum* defense against *B. thuringiensis* was also demonstrated [16]. The beetle populations differed in the specific sets and the expression dynamics of these genes, some of which were also differentially expressed upon pricking infection. For example, whereas OBP genes generally showed up-regulation 6 h after oral infection, Cro1 showed down-regulation of OBP genes after pricking infection.

Finally, *Osiris 18* and *19* were strongly up-regulated upon oral infection specifically in the Cro1 population while they were down-regulated in SB. Osiris comprises a family of highly conserved insect proteins of unknown function [47]. Interestingly, Osiris genes were recently shown to be up-regulated in honeybees upon oral infection with *Paenibacillus larvae* although developmental asynchrony could not be excluded as an indirect cause of differential expression [27]. Nevertheless, Osiris genes emerge as interesting targets for future studies of their potential role in defense against orally infecting pathogens.

### Gene expression upon pricking infection contains a strong signal of wounding

Pricking infection consists of wounding and delivery of bacteria into the hemocoel. Therefore, transcriptional responses to pricking infection resemble wounding responses, but show additional components. This is clearly visible from differentially expressed gene numbers (Figure 3) and the GO term enrichment analyses (Figure 4), where pricking infection resembles wounding, while oral infection shows clearly distinct patterns. The strong overlap of differentially expressed gene numbers and GO term enrichment between bacterial pricking (BttP) and sterile wounding as a pricking control (PC) shows that the wounding response makes up for a large part of the observed effects of bacterial pricking. Many genes overrepresented for both BttP and PC treatment were identified as cuticle related in response to the physical damage of pricking and potentially also the induced developmental asynchrony of both treatments compared to naive beetles.

Also regarding stress related genes, Cytochrome P450, odorant binding and developmental genes, the bacterial pricking and sterile wounding treatments show rather similar enrichment (Figure 5). However, a more detailed comparison of immune pathways also revealed genes that are up-regulated specifically after bacterial pricking but not in the pricking control, such as *Dif2*, *IMD* and *REL2* (Relish), which are central to the regulation of the two main immune-inducible pathways IMD and Toll; see Additional file 14. This might indicate that bacterial pricking (compared to wounding alone) leads to a stronger or more long-lasting activation of these pathways. While *IMD* is mostly involved in defense against Gram-negative bacteria, *Toll* is generally activated by Gram-positive bacteria and fungi (see [21] for a review on *Drosophila* immunity and [48] for *T. castaneum*). However, even though *Bacillus* is Gram-positive, it has DAP-type peptidoglycan, which is characteristic of Gram-negatives and activates the IMD pathway. Accordingly, we found both pathways activated after pricking infection with *B. thuringiensis*, and a large number of AMPs including *Attacin1, Attacin2, Cecropin2, Cecropin3, Defensin1, Defensin3, Lysozyme2* and *Lysozyme3* were strongly induced. In sum, our study clearly shows that sterile wounding is a necessary control to distinguish, which genes are involved in the wounding, and which are additionally relevant for combating the bacteria.

## Conclusions

Our study shows that different routes of infection lead to strongly divergent gene expression profiles in the host, and that it is therefore important to include the aspect of infection routes into studies of host-pathogen interactions. Importantly, host populations differed in their responses in particular to oral infection. This suggests a higher degree of phenotypic and genetic variance for responses to oral infection, as compared to the more ‘hard-wired’ responses to pricking infection. It thus also corroborates a recent finding that evolutionary adaptation was faster for resistance to oral as compared to pricking infection [3]. Host population differences emerge as an interesting field for future investigation, since a recently collected beetle population (Cro1) seems to react with more powerful immune reactions and to alleviate collateral damage, while a laboratory strain (SB) seems to have lost this flexibility. Resistance mechanisms showing strong diversity among populations are presumably evolving fast, and there is indeed some overlap of the population-specific resistance mechanisms identified in our study and the degree of adaptive evolution detected in immune system genes in *Drosophila* [49]. Moreover, we found indications that the more outbred population may show a reduced degree of adaptive developmental plasticity. Genetic diversity in the interaction of immunity with development might thus be a relevant field for future studies to advance our understanding of resistance in an ecological and evolutionary context.

## Methods

### Insects

Two populations of *T. castaneum* were examined in this study. As a standard laboratory strain San Bernardino (SB) was used. Furthermore, a wild type strain, Croatia 1 (Cro1), collected in June 2010 in Croatia, was included [4]. This strain was adapted to lab condition for more than 20 generations (18 months). Beetles were reared on flour (type 550) with 5% brewer yeast at 30°C with a 12/12 h light/dark cycle.

### Bacteria

*Bacillus thuringiensis bv. tenebrionis* (Btt) spores were purchased from the Bacillus Genetic Stock Center (BGSC) and subcloned five times on LB-Agar before a log-phase culture was used for glycerin stocks that were stored at −80°C.

### Infection experiment

Approximately 500 two weeks old adults of each strain were used for egg production for 24 h, respectively. Eggs were sieved of the flour with 280 *μ*m sieve and further cultivated under given standard conditions for another 10 days. Larvae were sieved of and individualized into 96 well plates containing flour and 5% yeast. After an additional time span of six days larvae of each strain were randomly assigned to the following treatments: Btt pricking (BttP), Btt oral (BttO), pricking control (PC), naïve control (NC). For analysis of differential RNA expression over time we choose 2 time points (6 h; 18 h) to sample total RNA. Each treatment was done with both beetle strains and 3 replicates with 32 larvae each (2 beetle strains × 4 treatments × 2 time points × 3 replicates = 48 samples). For BttP, bacteria were grown from a glycerin stock over night for 15 h in 50 ml standard LB-Media and centrifuged for 15 min, 5000×g at 4°C and washed with PBS twice. After last wash the pellet was resuspended in 2 ml PBS and counted in a Thoma counting chamber to adjust the concentration to 1 × 10^10^ per ml (LD_20_). For infection the larva was pricked dorsally between 1^st^ and 2^nd^ integument into the main vessel with a sterile dissecting needle (Ø10 *μm*) that was dipped into either the bacteria solution (BttP) or into PBS (PC). After pricking, larvae were individualized in 96 well plates containing flour and 5% yeast until sampling. For BttO spore production, preparation of spore-containing diet and the infection protocol were done as previously described [4]. Briefly, spores were produced and the suspension was centrifuged at 2880 × g at RT for 15 min, washed once, resuspended in PBS and counted using a Thoma counting chamber. Freshly produced spores were mixed with the beetle diet (0.15 g of flour with yeast/mL) in a concentration of 1 × 10^9^ spores per ml (LD_20_) of diet. 40 *μ*l of spore-containing liquid diet was pipetted into each well of a 96 well plate and dried overnight at 50°C. For infection, larvae were exposed to the spore-containing diet for three hours after which they were transferred to the spore-free diet until sampling. This was done since longer exposure does not contribute to further mortality to a higher extent [4]. Control larvae were treated in the same way. Larvae were sampled 6 and 18 h after the initial exposure had started (zero time point being the time point when larva was placed on the disc), since the majority of larvae usually start feeding within minutes after being placed on the discs (personal observation). For each NC sample 16 larvae were kept under the same conditions as in BttP and PC and another 16 larvae were kept under the same conditions as in BttO without infection. For RNA isolation, 32 larvae of each treatment were pooled for one sample after 6 h and 18 h, respectively. Samples were shock frozen in liquid nitrogen and stored at −80°C. Total RNA from frozen beetles was isolated using mirVana ^TM^ miRNA Isolation Kit (Ambion) according to the instructions of the manufacturer; for an overview of the infection experiment see Figure 1.

### Library preparation, sequencing and mapping

The libraries were created with the Illumina TruSeq RNA Sample Prep Kit v2. After cluster generation with the TruSeq PE Cluster Kit v3 (cBot –HS) the sequencing was performed with the TruSeq SBS Kit v3 –HS (200 cycles) on the Illumina HiSeq 2000. Before the mapping, the paired end reads of length 101 bp were preprocessed in the following way: (1) adapter sequences were removed from the data set using SeqPrep [50], (2) reads that did not pass the internal Illumina quality filter were eliminated and (3) due to a non-uniform distribution of nucleotides at the beginning of the reads – a problem naturally occurring caused by the random hexamer priming step in cDNA generation [51] – the first 13 base pairs of every read were trimmed using the FASTX-toolkit, version 0.0.13 [52]. Afterwards, the trimmed and filtered paired-end reads were mapped against the *T. castaneum* reference genome, version 3.0, downloaded from Beetlebase [53–55], making use of the RNA-seq read mapper Tophat, version 2.0.8b [56], with Bowtie version 2.1.0 [57]. Setting option -G, Tophat was also supplied with the *T. castaneum* genome annotation in gtf format, downloaded from the iBeetle webpage in June 2013 (“AUGUSTUS 2 prediction”) [58, 59]. iBeetle is an initiative of the group around Prof. Mario Stanke to enhance the current Beetlebase annotation of the *T. castaneum* genome by integrating data of various RNA sequencing experiments. In the “Beetle community”, this unofficial genome annotation is considered to be more accurate compared to the current official *T. castaneum* annotation from Beetlebase.

### Transcript assembly, library normalization and differential expression analysis

For every sample, transcript assembly and quantification have been carried out using cufflinks from Cufflinks, version 2.1.1, with option -G which tells Cufflinks to use the supplied reference gft annotation file from iBeetle and with option --upper-quartile-norm [58–60]. This latter option entails an upper quartile (75th percentile) normalization within each library and improves sensitivity without loss of specificity for differential expression calls [61]. Then the Cufflinks utility cuffmerge from Cufflinks was run to merge these assemblies into a comprehensive transcriptome. Significant changes in transcript expression between two different conditions were then detected employing the Cufflinks tool cuffdiff with option --upper-quartile-norm and the default q-value cutoff of 0.05. The utility cuffdiff performs this upper-quartile normalization across the whole set of samples and thus accounts for differences in library size and sequencing depth. To assess the distributions of upper-quartile normalization based FPKM (Fragments Per Kilobase of exon per Million fragments mapped) values across samples, a boxplot was produced using the R package cummeRbund [62]; see Additional file 15.

### Principal component analyses

The principle component analyses were based on the normalized FPKM values calculated with cuffdiff and performed with the method pca in R (package “labdsv”) using the covariance matrix [63]. The scores of the first two dimensions were plotted with the R function plot.

### Gene annotation and functional analysis

Genes of interest were annotated in two different ways. First, iBeetle genes were matched against the official Beetlebase gene identifiers by using a best reciprocal Blast hits based association table kindly provided by the iBeetle consortium [58, 59]. Using the official Beetlebase gene identifiers, gene descriptions, Gene Ontology (GO) terms [64] and InterPro attributes were downloaded from the EnsemblMetazoa database, release 17 [65]. In order to further improve the annotation of iBeetle genes, especially for those without a best reciprocal Blast hit among the Beetlebase gene identifiers, peptide sequences for genes of interest were downloaded from the iBeetle webpage and were blasted against the nr protein database using blastp from Blast2GO, version 2.6.6, and were then mapped and annotated with the Blast2GO default parameters [58, 59, 66]. Additionally, the Blast2GO InterProScan was applied and the InterProScan GOs were merged to the annotation [67].

### Overrepresentation analysis of GO terms and TermLogos

In order to detect GO terms assigned by Blast2GO that are overrespresented in a gene subsample of interest against all *T. castaneum* genes as background, the one-tailed Fisher’s Exact Test implemented in Blast2GO with a Benjamini Hochberg corrected p-value cutoff of 0.05 was applied [66]. Similarly to [12], using a scaling factor of *S*=| log10(p-value)| and a color scheme of length three to differentiate between the three different ontologies “biological process”, “cellular component” and “molecular function”, a TermLogo was generated making use of the web server Wordle ^*TM*^. The resulting word cloud thus visualizes GO terms that are enriched in a subsample of interest against all *T. castaneum* genes as background such that the color within the TermLogo represents one of the three ontologies and the font size the significance of every GO term according to the overrepresentation analysis: the larger the font, the smaller the corresponding p-value [12].

### Overrepresentation analysis of gene categories

Based on a list of 388 *T. castaneum* immunity-related genes published by [14] (of which 305 genes could be matched to genes predicted by iBeetle; of those genes, 303 genes could be found among assembled transcripts in our RNA-seq data set) separated into the categories “recognition”, “extracellular signaling”, “intracellular signaling” (with subcategories “Toll Pathway”, “IMD pathway”, “JNK pathway” and “JAK-STAT pathway”) and “execution” (with subcategories “PO”, “ROS”, “AMP”, “cellular responses”), we tested whether any of these (sub-) categories is overrepresented in the list of either significantly up- or downregulated genes (q-value cutoff: 0.05) for every pairwise Cufflinks differential expression result using Fisher’s Exact Test. For example, we tested whether there is a significant overrepresentation of “AMP” genes in the list of significantly upregulated genes resulting from the comparison of Cro1:BttO:6h vs. Cro1:NC:6h. The resulting p-values were corrected for multiple testing according to the Benjamini Hochberg procedure. Afterwards, the adjusted p-values were visualized using the function heatmap.2 from the R library “gplots” [68]. Apart from the list of immune genes by [14], this enrichment analysis was also applied to additional genes sets. We defined a set of cuticle genes based on the *T. castaneum* GO annotation downloaded from the EnsemblMetazoa database, release 17 by extracting only those genes with the GO term “structural constituent of cuticle”. The resulting list of cuticle genes contains 120 genes of which 111 could be linked to iBeetle gene predictions. A further list of stress-responsive genes involving heat shock genes was defined by taking all *T. castaneum* genes that contain the string “stress” or “heat shock” in at least one GO term downloaded from the EnsemblMetazoa database. Of this list of 58 stress genes, 51 could be matched to iBeetle predictions. Furthermore, a set of P450 genes was defined by extracting all *T. castaneum* genes with the term “P450” in the corresponding EnsemblMetazoa gene’s description (120 genes of which 89 have an iBeetle equivalent). Similarly, the list of odorant binding genes is based on extracting all *T. castaneum* genes with the GO term “odorant binding” resulting in 262 genes of which 103 could be linked to iBeetle predictions. A list of developmental genes was defined based on Table S11 from [8] containing 442 “selected developmental genes” of which 397 genes could be both matched to genes predicted by iBeetle and found among assembled transcripts in our RNA-seq dataset. After applying Fisher’s Exact Test to these gene lists, the resulting p-values were corrected for multiple testing and visualized in heatmaps as stated above.

## Availability of supporting data

The data sets supporting the results of this article are available in the National Center for Biotechnology Information (NCBI) Sequence Read Archive (SRA), accession number SRP033773.

## Electronic supplementary material

Additional file 1: **Table S1.** Numbers of reads. Illumina Reads were preprocessed by removing adapter sequences, by eliminating reads that did not pass the internal Illumina quality filter and by trimming the first 13 base pairs of every read using SeqPrep and FASTX [50, 52]. Afterwards, preprocessed reads were mapped against the *T. castaneum* reference genome, version 3.0, using Tophat [56]; R is short for “Replicate”. (PDF 24 KB)

Additional file 2: **Table S2.** Numbers of differentially expressed genes. The numbers of significantly up- and downregulated genes result from differential expression analyses for every treatment against its naïve control using Cufflinks with the default q-value cutoff of 0.05 [60]. (PDF 19 KB)

Additional file 3: **Figure S1.** Principal component analysis for all samples 6 h after exposure. The analysis is based on the normalized FPKM values calculated with cuffdiff and has been performed with the R package “labdsv” [60, 63]. (PDF 5 KB)

Additional file 4: **Figure S2.** Principal component analysis for all samples 18 h after exposure. The analysis is based on the normalized FPKM values calculated with cuffdiff and has been performed with the R package “labdsv” [60, 63]. (PDF 5 KB)

Additional file 5: **Figure S3.** Venn diagrams of differentially expressed genes 18 h after infection. The sets of differentially expressed genes result from differential expression analyses for every treatment against its naïve control using Cufflinks with the default q-value cutoff of 0.05 [60]. Venn diagram of significantly **A)** upregulated genes in all combinations of the populations Cro1 and SB and the treatments BttO and BttP, **B)** downregulated genes in all combinations of the populations Cro1 and SB and the treatments BttO and BttP, **C)** upregulated genes in all combinations of the populations Cro1 and SB and the treatments BttP and PC, **D)** downregulated genes in all combinations of the populations Cro1 and SB and the treatments BttP and PC. (PDF 530 KB)

Additional file 6: **Table S3.** TOP 30 overrepresented GO terms in sets of differentially expressed genes. For the detection of overrepresented GO terms associated with a gene subsample of interest, the one-tailed Fisher’s Exact Test implemented in Blast2GO with a FDR cutoff (Benjamini Hochberg corrected p-value) of 0.05 was applied with all *T. castaneum* genes as background [66]. On the six sheets the TOP 30 overrepresented GO terms for significantly upregulated genes in BttO (BttO_up), downregulated genes in BttO (BttO_down), upregulated genes in BttP (BttP_up), downregulated genes in BttP (BttP_down), upregulated genes in PC (PC_up) and downregulated genes in PC (PC_down) are listed. (XLSX 39 KB)

Additional file 7: **Table S4.** Immune genes. The list contains all 303 immunity-related genes based on [14] labeled with one of the categories “recognition”, “extracellular signaling”, “intracellular signaling” or “execution”. It includes the official gene IDs (’TC######’), iBeetle IDs, information from [14] about “Gene_ID”, “Gene_name” and “Gene_family”, annotation from the EnsemblMetazoa database, release 17 [65] (“Description”, “GO Term Accession”, “GO Term Name”, “InterPro ID” and “InterPro Description”), information from the Cufflinks differential expression analysis about the locus of the corresponding transcript (“locus”) and the log2(fold_change) values (abbreviated by L2FC) of all twelve pairwise comparisons against their respective naïve controls as well as the corresponding q-values (abbreviated by q). (XLSX 161 KB)

Additional file 8: **Table S5.** Cuticle genes. The list contains all 111 cuticle genes, i. e. all genes with the GO term “structural constituent of cuticle” based on the *T. castaneum* GO annotation downloaded from the EnsemblMetazoa database, release 17 [65]. It includes the official gene IDs (’TC######’), iBeetle IDs, annotation from the EnsemblMetazoa database, release 17 [65] (“Gene Name”, “Description”, “GO Term Accession”, “GO Term Name”, “InterPro ID” and “InterPro Description”), information from the Cufflinks differential expression analysis about the locus of the corresponding transcript (“locus”) and the log2(fold_change) values (abbreviated by L2FC) of all twelve pairwise comparisons against their respective naïve controls as well as the corresponding q-values (abbreviated by q). (XLSX 85 KB)

Additional file 9: **Table S6.** Stress genes. The list contains all 51 stress related genes, i. e. all genes genes that contain the string “stress” or “heat shock” in at least one GO term based on the *T. castaneum* GO annotation downloaded from the EnsemblMetazoa database, release 17 [65]. It includes the official gene IDs (’TC######’), iBeetle IDs, annotation from the EnsemblMetazoa database, release 17 [65] (“Gene Name”, “Description”, “GO Term Accession”, “GO Term Name”, “InterPro ID” and “InterPro Description”), information from the Cufflinks differential expression analysis about the locus of the corresponding transcript (“locus”) and the log2(fold_change) values (abbreviated by L2FC) of all twelve pairwise comparisons against their respective naïve controls as well as the corresponding q-values (abbreviated by q). (XLSX 70 KB)

Additional file 10: **Table S7.** P450 genes. The list contains all 89 cytochrome P450 genes, i. e. all genes with the term “P450” in the corresponding EnsemblMetazoa gene’s description [65]. It includes the official gene IDs (’TC######’), iBeetle IDs, annotation from the EnsemblMetazoa database, release 17 [65] (“Gene Name”, “Description”, “GO Term Accession”, “GO Term Name”, “InterPro ID” and “InterPro Description”), information from the Cufflinks differential expression analysis about the locus of the corresponding transcript (“locus”) and the log2(fold_change) values (abbreviated by L2FC) of all twelve pairwise comparisons against their respective naïve controls as well as the corresponding q-values (abbreviated by q). (XLSX 84 KB)

Additional file 11: **Table S8.** Differential expression results. The table contains twelve sheets, each with a list of differentially expressed genes for a treatment against its naïve control based on Cufflinks analyses with a default q-value cutoff of 0.05 [60]. The first column gives the Cufflinks Test ID followed by the iBeetle ID, the corresponding official gene ID (’TC######’) based on a best reciprocal Blast hits based association table [58, 59], annotation from the EnsemblMetazoa database, release 17 [65] (“Gene Name”, “Description”, “GO Term Accession”, “GO Term Name”, “InterPro ID” and “InterPro Description”), information from the Cufflinks differential expression analysis about the locus (“locus”), the sample names, the FPKM values of each sample (“value_1” and “value_2”), the log2(fold_change) value, the value of the test statistic, the p-value and the FDR-adjusted q-value of the test statistic. (XLSX 2 MB)

Additional file 12: **Table S9.** Odorant binding genes. The list contains all 103 odorant binding genes, i. e. all genes with the GO term “odorant binding” in the EnsemblMetazoa database, release 17 [65]. It includes the official gene IDs (’TC######’), iBeetle IDs, annotation from the EnsemblMetazoa database, release 17 [65] (“Gene Name”, “Description”, “GO Term Accession”, “GO Term Name”, “InterPro ID” and “InterPro Description”), information from the Cufflinks differential expression analysis about the locus of the corresponding transcript (“locus”) and the log2(fold_change) values (abbreviated by L2FC) of all twelve pairwise comparisons against their respective naïve controls as well as the corresponding q-values (abbreviated by q). (XLSX 84 KB)

Additional file 13: **Table S10.** Developmental genes. The list contains 397 developmental genes based on [8]. It includes the official gene IDs (’TC######’), iBeetle IDs, information from [8] about the “Gene Name”, annotation from the EnsemblMetazoa database, release 17 [65] (“Description”, “GO Term Accession”, “GO Term Name”, “InterPro ID” and “InterPro Description”), information from the Cufflinks differential expression analysis about the locus of the corresponding transcript (“locus”) and the log2(fold_change) values (abbreviated by L2FC) of all twelve pairwise comparisons against their respective naïve controls as well as the corresponding q-values (abbreviated by q). (XLSX 195 KB)

Additional file 14: **Figure S4.** Regulation of the Toll and IMD pathway for PC and BttP 6 h after infection. Illustrated are the Toll and IMD pathways after [14] for the two populations of *T. castaneum* Cro1 and SB and for the two infection methods PC and BttRed, P., indicates significant upregulation and blue significant downregulation of respective genes. The corresponding official gene IDs (’TC######’) are specified next to the genes. When different gene family members are both significantly up- and downregulated, genes are presented in a purple manner and the colors of the corresponding official gene ID indicate in more detail which isoforms are up- and which ones are downregulated. Only effectors that are differentially expressed 6 h after infection in at least one treatment are indicated with their respective official gene IDs; see also Additional file 7. (PDF 631 KB)

Additional file 15: **Figure S5.** Boxplot of base-10 logarithmic FPKM values for all individual treatments produced by cummeRbund [62]. FPKM values result from running both Cufflinks utilities cufflinks and cuffdiff with option --upper-quartile-norm, i. e. an upper quartile normalization has been applied within as well as between libraries. (PDF 131 KB)
