# Supplementary material for: Infection routes matter in population-specific responses of the red flour beetle to the entomopathogen Bacillus thuringiensis
Source: BMC Genomics. 2014 Jun 7;15(1):445. doi: 10.1186/1471-2164-15-445 (PMC4079954; doi:10.1186/1471-2164-15-445)
Supplement: Supplementary file 1 — Additional file 1: Table S1. Numbers of reads. Illumina Reads were preprocessed by removing adapter sequences, by eliminating reads that did not pass the internal Illumina quality filter and by trimming the first 13 base pairs of every read using SeqPrep and FASTX [50, 52]. Afterwards, preprocessed reads were mapped against the T. castaneum reference genome, version 3.0, using Tophat [56]; R is short for “Replicate”. (PDF 24 KB) [file 12864_2014_6181_MOESM1_ESM.pdf]

library-ID	sample-ID	Treatment	# reads	# filtered reads	# mapped reads
B0986	1	SB:PC:6h:R2	58,930,526	51,393,312	37,087,923
B0987	2	Cro1:BttP:6h:R1	63,554,868	55,826,596	42,305,204
B0988	3	Cro1:BttP:18h:R2	52,145,748	46,075,983	34,633,441
B0989	4	SB:BttP:6h:R3	43,652,524	38,470,969	27,599,500
B0990	5	Cro1:BttP:6h:R2	49,062,398	43,194,535	32,347,045
B0991	6	SB:BttP:6h:R1	48,353,724	42,256,319	30,221,376
B0992	7	SB:PC:18h:R1	56,341,822	49,068,093	35,395,370
B0993	8	Cro1:PC:18h:R1	54,752,928	47,651,473	35,609,743
B0994	9	Cro1:BttP:6h:R3	53,080,992	46,950,137	36,599,240
B0995	10	SB:BttP:18h:R3	71,614,874	62,677,338	47,884,394
B0996	11	Cro1:PC:18h:R3	68,297,014	59,070,087	46,412,898
B0997	12	Cro1:PC:18h:R2	65,729,278	57,243,628	44,003,442
B0998	13	Cro1:PC:6h:R2	65,561,638	57,845,033	45,742,140
B0999	14	SB:BttP:6h:R2	51,719,344	45,476,819	34,878,699
B1000	15	SB:PC:18h:R2	52,033,642	46,044,570	34,085,307
B1001	16	SB:PC:6h:R3	58,490,596	51,068,139	38,668,558
B1002	17	Cro1:PC:6h:R1	58,394,870	50,225,428	36,773,057
B1003	18	SB:BttP:18h:R1	59,384,574	50,470,949	35,670,541
B1004	19	SB:PC:6h:R1	56,194,390	47,984,390	33,346,443
B1005	20	Cro1:PC:6h:R3	54,428,264	46,519,837	34,127,946
B1006	21	Cro1:BttP:18h:R3	69,126,244	58,771,133	42,665,874
B1007	22	Cro1:BttP:18h:R1	65,994,168	56,240,230	41,125,573
B1008	23	SB:PC:18h:R3	56,119,480	47,802,573	33,659,916
B1009	24	SB:BttP:18h:R2	71,187,456	60,502,219	41,297,486
B1010	25	SB:BttO:18h:R1	66,192,768	51,577,405	35,356,280
B1011	26	Cro1:NC:18h:R3	66,988,490	53,068,282	36,707,255
B1012	27	SB:BttO:6h:R2	59,890,922	47,703,119	31,754,117
B1013	28	SB:NC:18h:R3	47,525,822	37,882,833	26,160,660
B1014	29	SB:NC:18h:R2	55,839,022	44,246,841	30,065,203
B1015	30	Cro1:BttO:6h:R3	59,391,124	46,426,042	31,784,114
B1016	31	Cro1:NC:18h:R1	80,501,492	62,936,066	43,518,259
B1017	32	Cro1:NC:6h:R2	71,296,796	55,176,590	38,484,153
B1018	33	SB:NC:18h:R1	62,071,238	44,002,301	24,168,233
B1019	34	SB:BttO:18h:R3	69,915,778	49,052,910	27,513,039
B1020	35	SB:NC:6h:R1	58,678,368	40,781,466	23,073,273
B1021	36	SB:NC:6h:R2	59,157,112	41,427,726	22,208,908
B1022	37	Cro1:BttO:6h:R2	84,491,760	60,065,192	34,059,696
B1023	38	Cro1:NC:18h:R2	59,591,652	42,131,298	24,084,598
B1024	39	SB:BttO:6h:R3	47,820,078	33,933,127	18,440,046
B1025	40	Cro1:BttO:6h:R1	53,036,586	37,332,453	20,864,319
B1026	41	Cro1:NC:6h:R1	63,119,418	54,718,223	36,180,553
B1027	42	Cro1:BttO:18h:R2	62,509,892	53,633,487	35,969,366
B1028	43	SB:BttO:6h:R1	56,358,552	48,468,355	32,936,168
B1029	44	Cro1:NC:6h:R3	54,232,976	46,851,868	30,260,767
B1030	45	Cro1:BttO:18h:R1	58,970,634	50,413,995	35,232,240
B1031	46	SB:BttO:18h:R2	65,722,126	56,291,001	39,369,070
B1032	47	Cro1:BttO:18h:R3	58,291,568	49,810,145	35,044,226
B1033	48	SB:NC:6h:R3	66,223,130	56,660,510	37,717,148
All Reads			2,891,968,666	2,383,421,026	1,653,092,808
