# Supplementary material for: Infection routes matter in population-specific responses of the red flour beetle to the entomopathogen Bacillus thuringiensis
Source: BMC Genomics. 2014 Jun 7;15(1):445. doi: 10.1186/1471-2164-15-445 (PMC4079954; doi:10.1186/1471-2164-15-445)
Supplement: Supplementary file 2 — Additional file 2: Table S2. Numbers of differentially expressed genes. The numbers of significantly up- and downregulated genes result from differential expression analyses for every treatment against its naïve control using Cufflinks with the default q-value cutoff of 0.05 [60]. (PDF 19 KB) [file 12864_2014_6181_MOESM2_ESM.pdf]

Comparison Pair	# upregulated genes	# downregulated genes	Sum
Cro1:BttO:6h vs. Cro1:NC:6h	671	681	1352
Cro1:BttO:18h vs. Cro1:NC:18h	552	511	1063
SB:BttO:6h vs. SB:NC:6h	460	784	1244
SB:BttO:18h vs. SB:NC:18h	102	191	293
Cro1:BttP:6h vs. Cro1:NC:6h	811	1225	2036
Cro1:BttP:18h vs. Cro1:NC:18h	590	575	1165
SB:BttP:6h vs. SB:NC:6h	551	964	1515
SB:BttP:18h vs. SB:NC:18h	284	225	509
Cro1:PC:6h vs. Cro1:NC:6h	651	935	1586
Cro1:PC:18h vs. Cro1:NC:18h	501	607	1108
SB:PC:6h vs. SB:NC:6h	498	582	1080
SB:PC:18h vs. SB:NC:18h	255	249	504
