# Supplementary figures and images for: Infection routes matter in population-specific responses of the red flour beetle to the entomopathogen Bacillus thuringiensis
Source: BMC Genomics. 2014 Jun 7;15(1):445. doi: 10.1186/1471-2164-15-445 (PMC4079954; doi:10.1186/1471-2164-15-445)

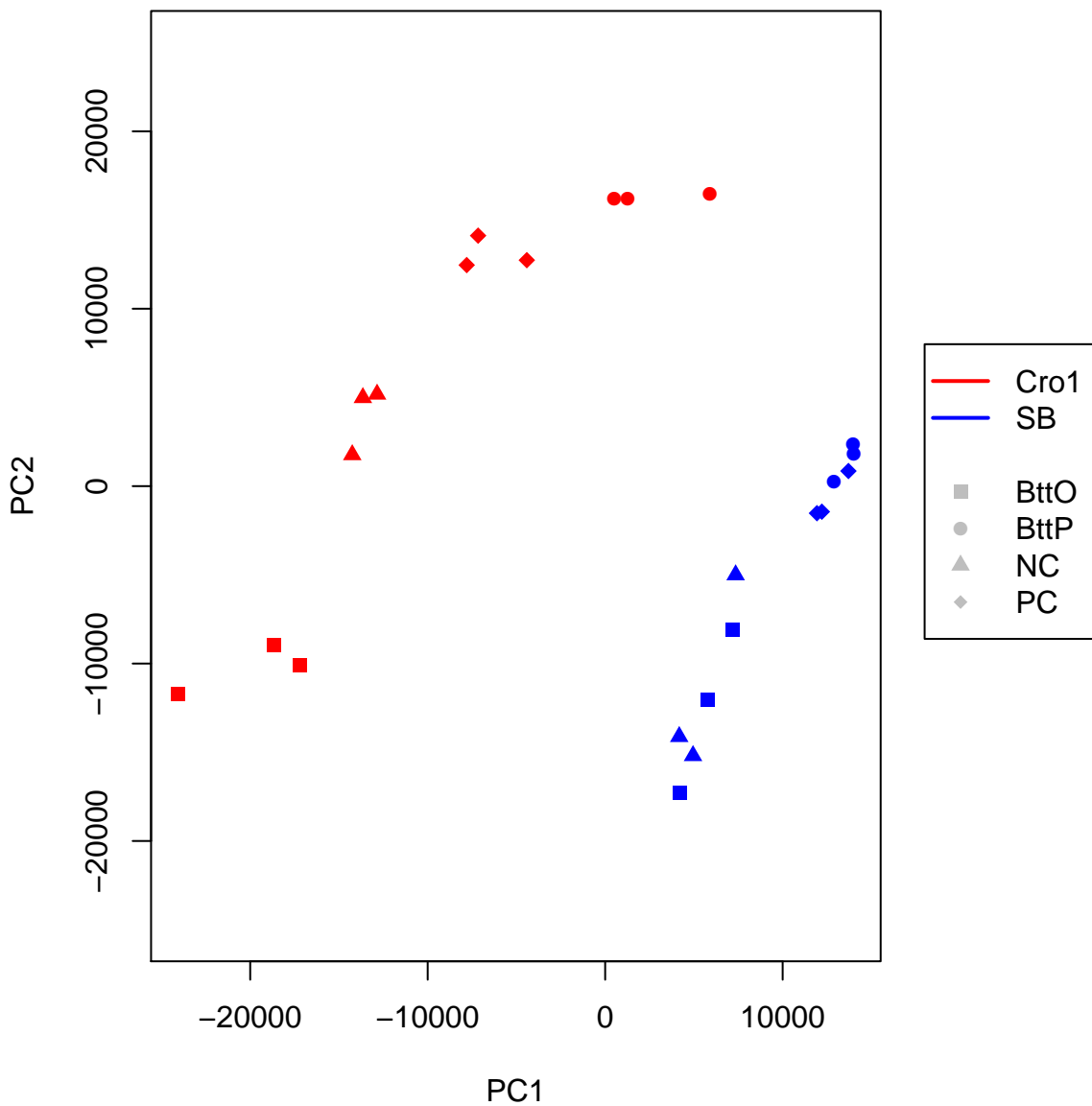

Supplement: Supplementary file 3 — Additional file 3: Figure S1. Principal component analysis for all samples 6 h after exposure. The analysis is based on the normalized FPKM values calculated with cuffdiff and has been performed with the R package “labdsv” [60, 63]. (PDF 5 KB) [file 12864_2014_6181_MOESM3_ESM.pdf]

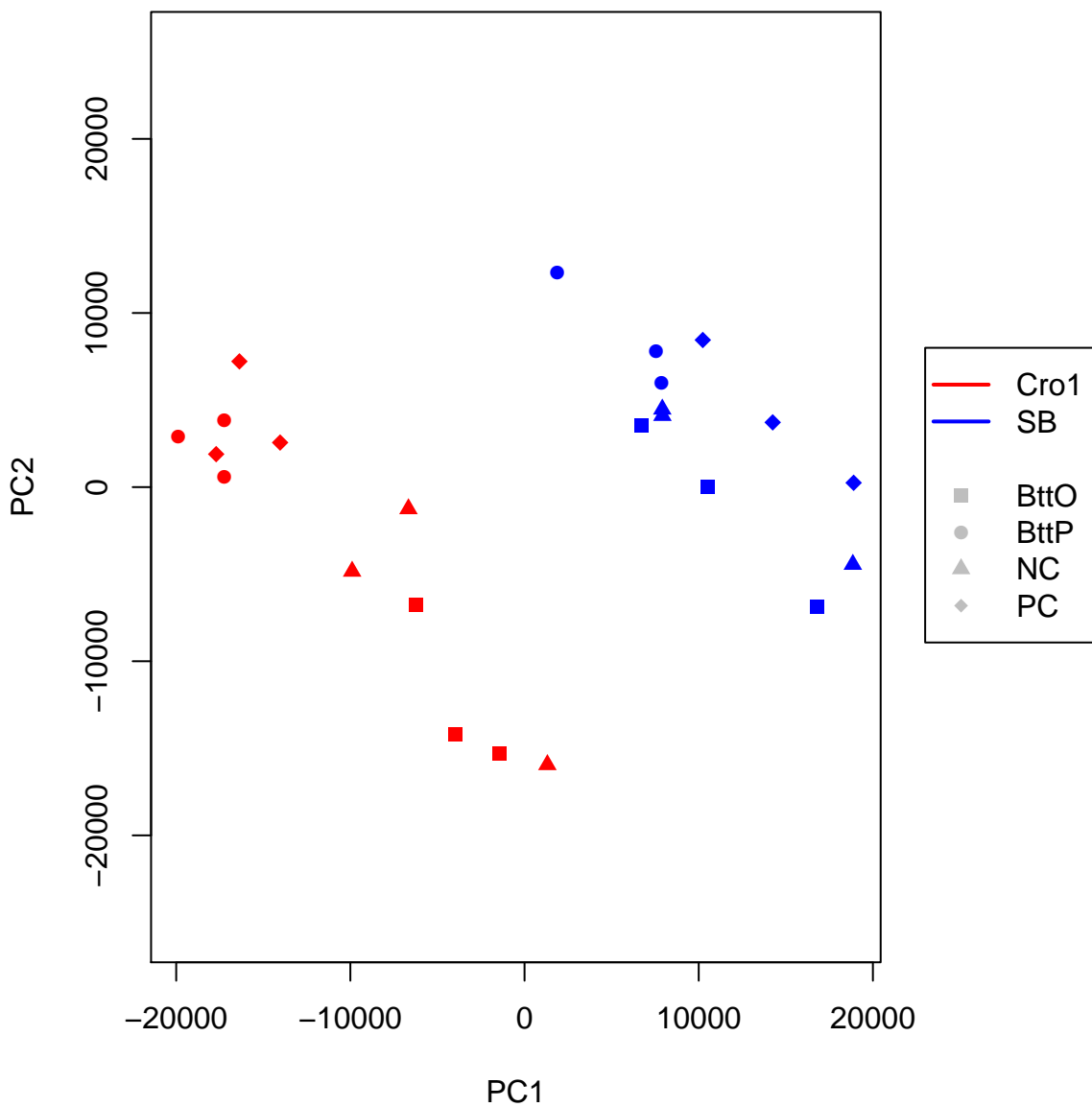

Supplement: Supplementary file 4 — Additional file 4: Figure S2. Principal component analysis for all samples 18 h after exposure. The analysis is based on the normalized FPKM values calculated with cuffdiff and has been performed with the R package “labdsv” [60, 63]. (PDF 5 KB) [file 12864_2014_6181_MOESM4_ESM.pdf]

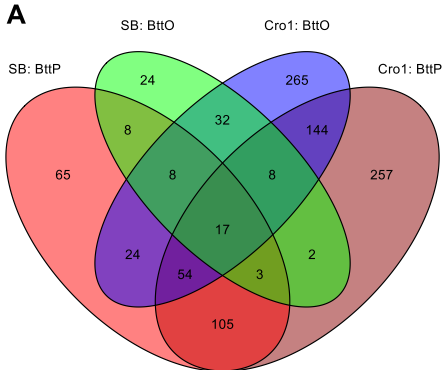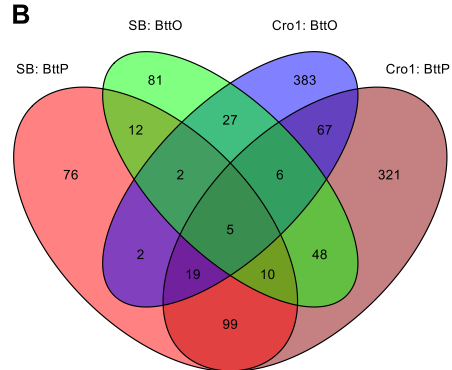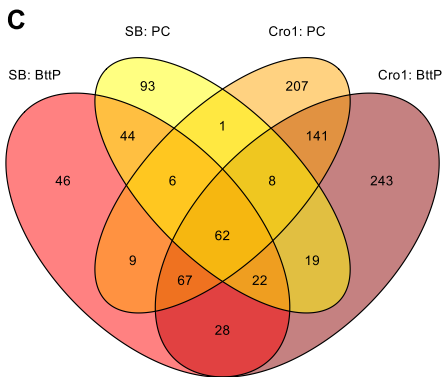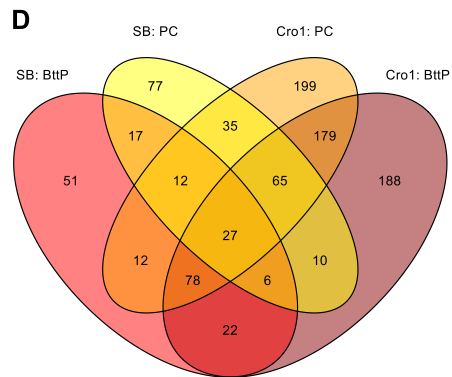

Supplement: Supplementary file 5 — Additional file 5: Figure S3. Venn diagrams of differentially expressed genes 18 h after infection. The sets of differentially expressed genes result from differential expression analyses for every treatment against its naïve control using Cufflinks with the default q-value cutoff of 0.05 [60]. Venn diagram of significantly A) upregulated genes in all combinations of the populations Cro1 and SB and the treatments BttO and BttP, B) downregulated genes in all combinations of the populations Cro1 and SB and the treatments BttO and BttP, C) upregulated genes in all combinations of the populations Cro1 and SB and the treatments BttP and PC, D) downregulated genes in all combinations of the populations Cro1 and SB and the treatments BttP and PC. (PDF 530 KB) [file 12864_2014_6181_MOESM5_ESM.pdf]

# Infection

## Pricking Control (PC)

## Pricking Infection (BttP)

Cro1

*T. castaneum* population

SB

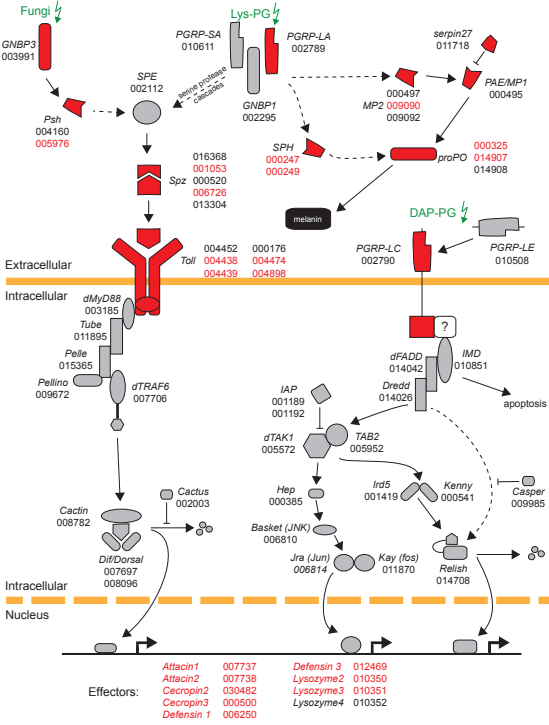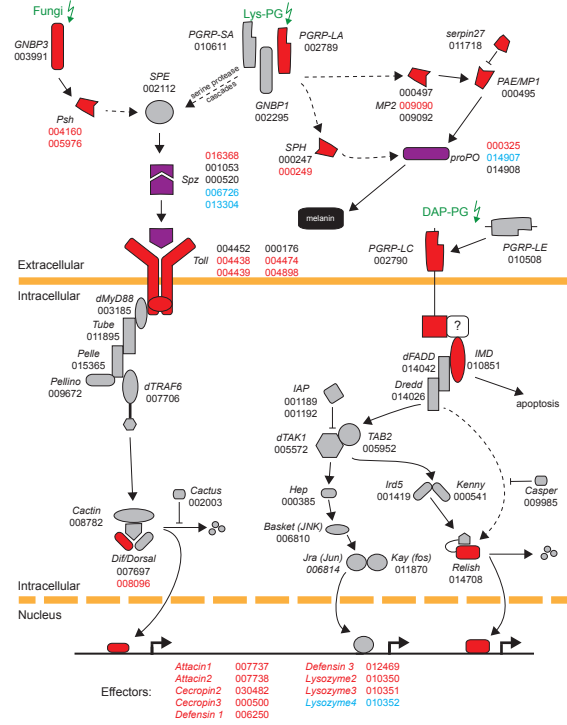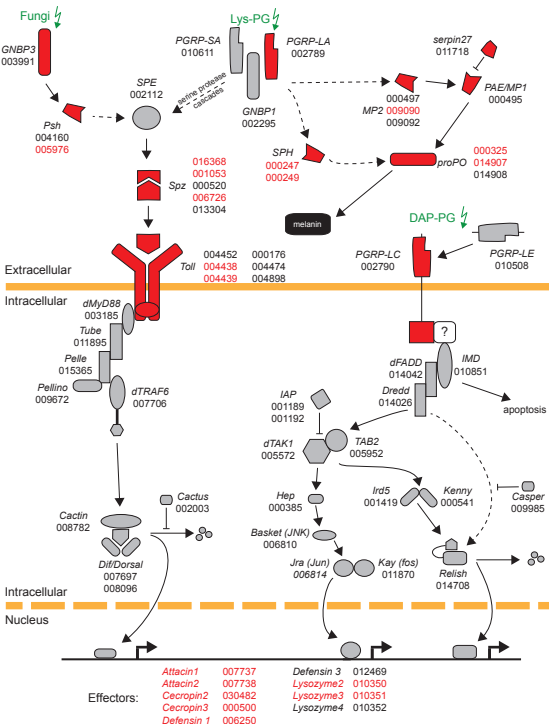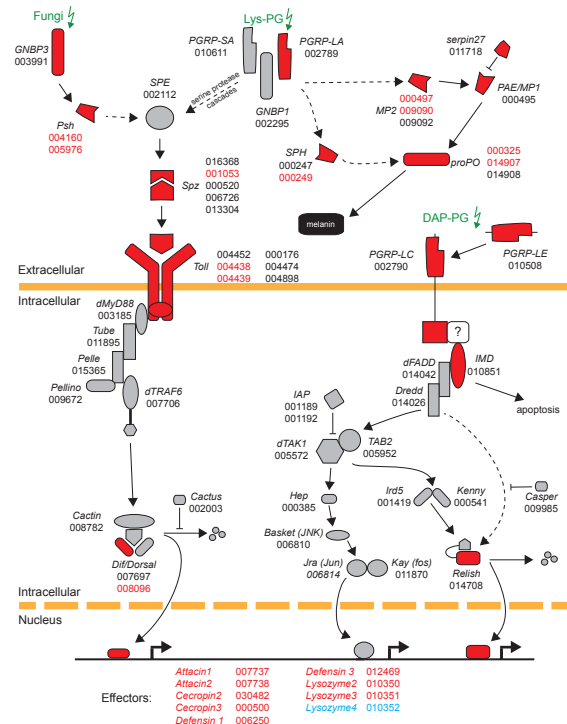

Supplement: Supplementary file 14 — Additional file 14: Figure S4. Regulation of the Toll and IMD pathway for PC and BttP 6 h after infection. Illustrated are the Toll and IMD pathways after [14] for the two populations of T. castaneum Cro1 and SB and for the two infection methods PC and BttRed, P., indicates significant upregulation and blue significant downregulation of respective genes. The corresponding official gene IDs (’TC######’) are specified next to the genes. When different gene family members are both significantly up- and downregulated, genes are presented in a purple manner and the colors of the corresponding official gene ID indicate in more detail which isoforms are up- and which ones are downregulated. Only effectors that are differentially expressed 6 h after infection in at least one treatment are indicated with their respective official gene IDs; see also Additional file 7. (PDF 631 KB) [file 12864_2014_6181_MOESM14_ESM.pdf]

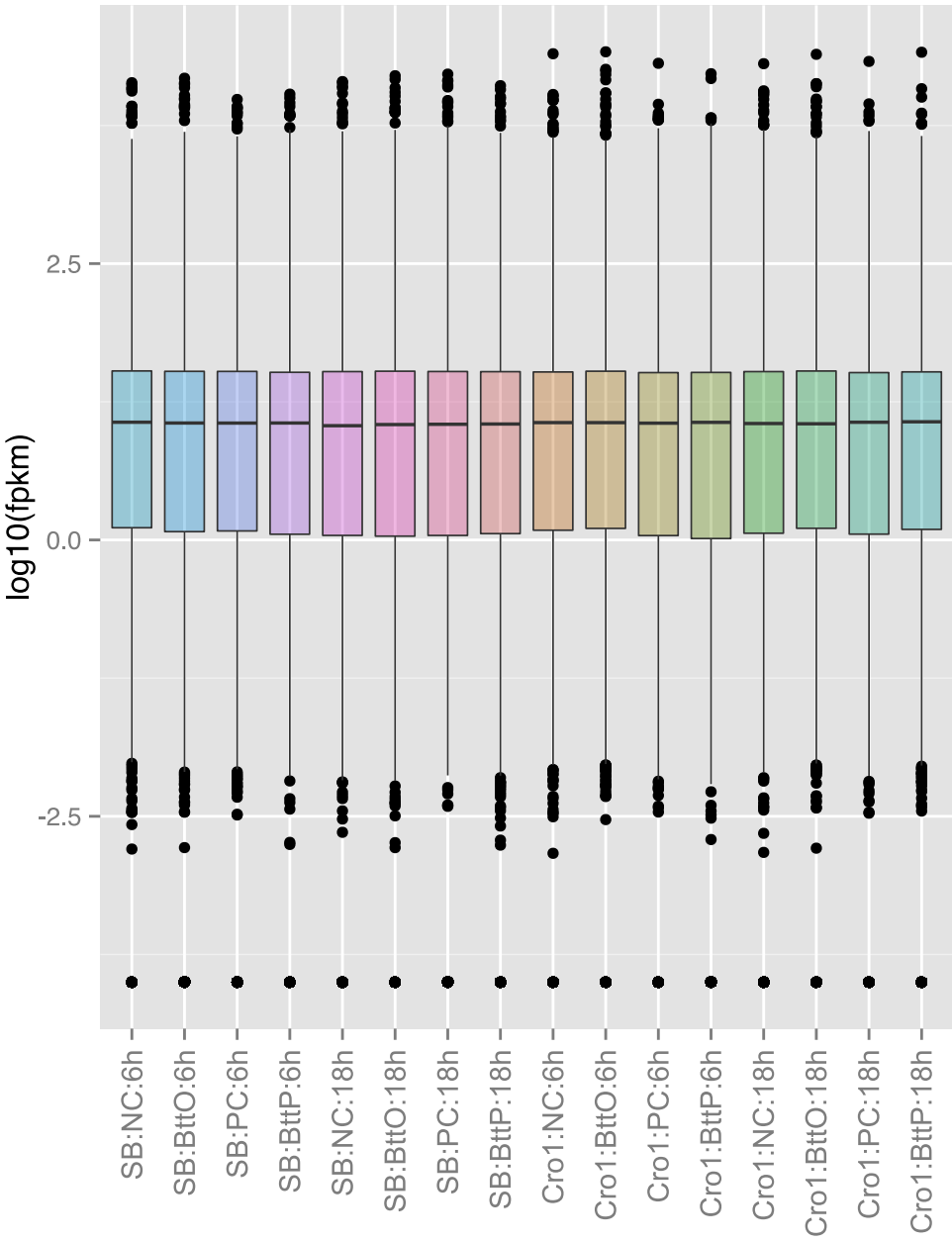

Supplement: Supplementary file 15 — Additional file 15: Figure S5. Boxplot of base-10 logarithmic FPKM values for all individual treatments produced by cummeRbund [62]. FPKM values result from running both Cufflinks utilities cufflinks and cuffdiff with option --upper-quartile-norm, i. e. an upper quartile normalization has been applied within as well as between libraries. (PDF 131 KB) [file 12864_2014_6181_MOESM15_ESM.pdf]
